# CD133-directed CAR T-cells for MLL leukemia: on-target, off-tumor myeloablative toxicity

**DOI:** 10.1038/s41375-019-0418-8

**Published:** 2019-02-18

**Authors:** Clara Bueno, Talia Velasco-Hernandez, Francisco Gutiérrez-Agüera, Samanta Romina Zanetti, Matteo L. Baroni, Diego Sánchez-Martínez, Oscar Molina, Adria Closa, Antonio Agraz-Doblás, Pedro Marín, Eduardo Eyras, Ignacio Varela, Pablo Menéndez

**Affiliations:** 1 Josep Carreras Leukemia Research Institute and Department of Biomedicine, School of Medicine, University of Barcelona, Barcelona, Spain; 2 Pompeu Fabra University, Barcelona, Spain; 3 Instituto de Biomedicina y Biotecnología de Cantabria (CSIC-UC-Sodercan), Departamento de Biología Molecular, Universidad de Cantabria, Santander, Spain; 4 Hematology Department, Hospital Clínico de Barcelona, Barcelona, Spain; 5 Instituciò Catalana de Recerca i Estudis Avançats (ICREA), Barcelona, Spain; 6 Centro de Investigación Biomédica en Red de Cáncer (CIBER-ONC), ISCIII, Barcelona, Spain

**Keywords:** Immunotherapy, Acute lymphocytic leukaemia

## To the Editor:

Chimeric antigen receptors (CARs) have undoubtedly revolutionized immunotherapy, especially in the B-cell acute lymphoblastic leukemia (ALL) arena where over 80% of complete remissions are observed in refractory/relapsed (R/R) B-cell ALL patients treated with CD19-directed CAR T-cells (CARTs) [1]. However, despite holding an unprecedented promise, several issues still have to be resolved before CARTs can be expanded to novel targets and/or malignancies or even provided as first-line treatment in B-cell ALL [2]. For instance, toxicities such as cytokine release syndrome and immune escape mechanisms including loss of the antigen under CART-mediated pressure remain major concerns, urging further research on the mechanisms underlying CARTs cytotoxicity.

In this sense, loss of CD19 antigen is frequently observed after CD19-directed CARTs therapy in B-cell ALL [3, 4], but is particularly common in MLL-rearranged (MLLr) B-cell ALL, an aggressive subtype of B-cell ALL (dismal in MLL-AF4+ infants) associated with lymphoid-to-myeloid lineage switch [3, 5, 6]. We read with interest the work recently published in Leukemia by Li et al. reporting a novel CAR targeting both CD19 and CD133 [7]. This study proposes to use a bi-specific CAR targeting both CD19 and CD133 antigens in a Boolean OR-gate approach for MLLr B-cell ALL as a strategy to avoid and treat CD19- relapses. The authors reasoned that CD133, encoded by *PROM1* gene, is a specific marker for MLLr leukemia because *PROM1* is an MLL target, especially in MLL-AF4 B-cell ALL [8–10]. They went on and performed in vitro assays showing than CD19/CD133 bi-specific CAR triggers robust cytotoxicity against CD19 + CD133 + and CD19-CD133+ B-cell lines [7], thus suggesting it may help in reducing subsequent lineage switch in MLLr B-cell ALL.

**Fig. 1:**
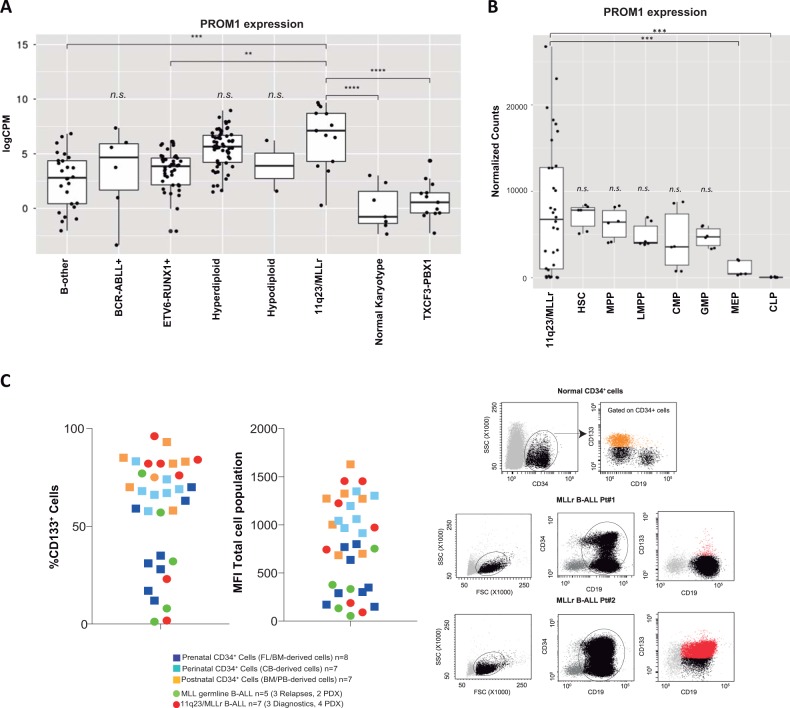
Characterization of CD133/*PROM1* expression in B-cell ALL and normal HSPCs. **a** Expression level of *PROM1* in the indicated cytogenetic subgroups of B-cell ALL (*n* = 170 patients at diagnosis) determined by RNA-seq represented in log2(CPM) scale, with CPM = counts per million [13]. **b** RNA-seq analysis comparing the expression of *PROM1* in 11q23/MLLr B-cell ALL (*n* = 29 patients) with that in distinct fractions of Lin-CD34 + CD38-CD19- non-lymphoid normal HSPCs (HSC hematopoietic stem cells, MPP multipotent progenitors, LMPP lymphoid-primed multipotent progenitors, CMP common myeloid progenitors, GMP granulocyte-monocyte progenitor, MEP megakaryocyte-erythroid progenitors) and in common lymphoid progenitors (CLP) [14]. Data shown as normalized counts. The boxes define the first and third quartiles. The horizontal line within the box represents the median. **c** Frequency (left) and mean fluorescence intensity (MFI, middle) of CD133+ BM blasts/cells in MLLr (*n* = 7) and non-MLL B-cell ALLs (*n* = 5) primary diagnostic/relapse samples or primografts (PDXs), and normal CD34+ HSPCs derived from FL (*n* = 8), CB (*n* = 7) and adult PB/BM (*n* = 7). Representative FACS dot plots for CD133 in normal CD34+ HSPCs (upper right) and BM samples from two independent MLLr B-cell ALL patients (bottom right)

A major drawback for CD133 as target in immunotherapy is its expression in hematopoietic stem and progenitor cells (HSPCs), which would likely exert “on-target off-tumor” myeloablative, life-threatening toxicity [11, 12]. Because B-cell ALL is molecularly heterogeneous and can be diagnosed during infancy, childhood and adulthood, we have characterized *PROM1*/CD133 expression in a large cohort of cytogenetically distinct B-cell ALL subgroups (*n* = 212 patients) as well as in different subpopulations of normal CD34+ HSPCs obtained across hematopoietic ontogeny from 22-weeks old human fetal liver (FL, prenatal), cord blood (CB, perinatal), and adult G-CSF-mobilized peripheral blood/bone marrow (PB/BM, postnatal). An initial analysis of publicly available RNA-seq data [13] from 170 diagnostic B-cell ALL patients confirmed that *PROM1* is overexpressed in patients with MLLr B-cell ALL, although its expression is not significantly higher than in other cytogenetic subgroups (Fig. 1a). We then analyzed PROM1 during HSPC development and observed that PROM1 is highly expressed in early normal hematopoietic stem cells (HSC) and multipotent progenitors (MPP) with its expression decreasing from the lymphoid-primed multipotent progenitors (LMPP) onwards with its expression being marginal at later stages of myeloid differentiation (megakaryocyte-erythroid progenitors, MEP) and common lymphoid progenitors (CLP) [14] (Fig. 1b). Importantly, 70% (22/32) of 11q23/MLLr B-cell patients (both MLL-AF4 and MLL-AF9) express equal (9/32) or lower (13/32) PROM1 levels that HSCs and MPPs, which raises doubts about the suitability of PROM1 as a target for B-cell ALL immunotherapy [15].

FACS clinical immunophenotyping provides a priori a more rapid and feasible clinically relevant diagnostic information than RNA-seq during the decision-making process. Thus, we next FACS-analyzed the expression of CD133 (*PROM1* gene product) in the cell surface of BM-derived primary blasts and primografts (PDXs) obtained from 11q23/MLLr (*n* = 7) and non-MLL (*n* = 5) B-cell ALL patients, and in comparison with healthy prenatal (22 weeks old FL), perinatal (CB) and adult (PB/BM) CD34+ HSPCs (Fig. 1c). Consistent with the RNA-seq data, the expression of CD133 in 11q23/MLLr blasts is intermingled with that observed in CD34+ HSPCs across hematopoietic ontogeny (Fig. 1c).

Our data demonstrates that *PROM1*/CD133 is similarly expressed between MLLr B-cell ALL primary blasts and normal non-lymphoid HSPCs across ontogeny, thus indicating that “on-target, off-tumor” toxic/myeloablative effects are likely to occur if used in a bi-specific CAR approach where CD133 antigen will be constantly targeted regardless of the co-expression of CD19 in the same cell. Our data therefore raises concerns about using CD133 as a target for MLLr B-cell ALL immunotherapy. An alternative to circumvent HSPC toxicity would be to engineer dual CAR T-cells with one CAR engaging an antigen (i.e., CD19) mediating T-cell activation and another CAR engaging a second antigen (i.e., CD133) mediating T-cell co-stimulation [16]. Unfortunately, although such a CD19/CD133 dual CAR might be likely safe due to its cytotoxicity being restrained only to cells co-expressing CD19 and CD133, its specific cytotoxic performance will be poor since not the entire MLLr B-cell ALL blast population is CD19 + CD133+ (Fig. 1c). Another alternative approach to prevent HSPC toxicity would be to have in place a potent molecular switch (i.e., iCas9) to eliminate CAR133-expressing T-cells as necessary [17]. Further long-term in vivo studies using both primary B-cell ALL cells and normal HSCPs remain to be conducted to elucidate the efficacy versus the myeloablative toxicity of a CAR CD133 [18, 19].

## Data Availability

All genomic data is already publicly available. A full data availability will be provided.
